# Tuberculosis treatment delays and associated factors within the Zimbabwe national tuberculosis programme

**DOI:** 10.1186/s12889-015-1437-7

**Published:** 2015-01-29

**Authors:** Kudakwashe C Takarinda, Anthony D Harries, Barnet Nyathi, Mkhokheli Ngwenya, Tsitsi Mutasa-Apollo, Charles Sandy

**Affiliations:** AIDS and TB Department, Zimbabwe Ministry of Health and Child Care, P. O Box CY 1122, Causeway, Harare Zimbabwe; International Union Against Tuberculosis and Lung Disease, Paris, France; Department of Clinical Research, London School of Hygiene and Tropical Medicine, London, UK; TB CARE Project, International Union Against Tuberculosis and Lung Disease, Harare, Zimbabwe

**Keywords:** Tuberculosis, Treatment, Delay, Zimbabwe

## Abstract

**Background:**

Delayed presentation of pulmonary TB (PTB) patients for treatment from onset of symptoms remains a threat to controlling individual disease progression and TB transmission in the community. Currently, there is insufficient information about treatment delays in Zimbabwe, and we therefore determined the extent of patient and health systems delays and their associated factors in patients with microbiologically confirmed PTB.

**Methods:**

A structured questionnaire was administered at 47 randomly selected health facilities in Zimbabwe by trained health workers to all patients aged ≥18 years with microbiologically confirmed PTB who were started on TB treatment and entered in the health facility TB registers between 01 January and 31 March 2013. Multivariate logistic regression was used to calculate adjusted odds ratios (aOR) and 95% confidence intervals (CIs) for associations between patient/health system characteristics and patient delay >30 days or health system delay >4 days.

**Results:**

Of the 383 recruited patients, 211(55%) were male with an overall median age of 34 years (IQR, 28-43). There was a median of 28 days (IQR, 21-63) for patient delays and 2 days (IQR, 1-5) for health system delays with 184 (48%) and 118 (31%) TB patients experiencing health system delays >30 days and health system delays >4 days respectively. Starting TB treatment at rural primary healthcare vs district/mission facilities [aOR 2.70, 95% CI 1.27-5.75, p = 0.01] and taking self-medication [aOR 2.33, 95% CI 1.23-4.43, p = 0.01] were associated with encountering patient delays. Associated with health system delays were accessing treatment from lower level facilities [aOR 2.67, 95% CI 1.18-6.07, p = 0.019], having a Gene Xpert TB diagnosis [aOR 0.21, 95% CI 0.07-0.66, p = 0.008] and >4 health facility visits prior to TB diagnosis [(aOR) 3.34, 95% CI 1.11-10.03, p = 0.045].

**Conclusion:**

Patient delays were longer and more prevalent, suggesting the need for strategies aimed at promoting timely seeking of appropriate medical consultation among presumptive TB patients. Health system delays were uncommon, suggesting a fairly efficient response to microbiologically confirmed PTB cases. Identified risk factors should be explored further and specific strategies aimed at addressing these factors should be identified in order to lessen patient and health system delays.

## Background

Tuberculosis remains an infectious disease of public health concern with the World Health Organisation (WHO) declaring it a global public health emergency in 1993 [1]. There has been a global increase in notified TB cases since then from an estimated 7-8 million cases to 9 million cases in 2013 [1]. In sub-Saharan Africa, the resurgence of TB has been attributed mostly to the HIV pandemic, and this has been felt most strongly in the southern part of the continent [2]. Providing treatment with anti-tuberculosis drugs through the directly observed treatment short course (DOTS) has greatly assisted most national tuberculosis control programmes in curing TB, and as a result it is estimated that from 2000 to 2013, 37 million lives have been saved [1]. However, the continued spread of TB can partly be attributed to delayed or undiagnosed TB and this in turn has also been shown to be associated with increased mortality [3].

The severity of disease and the likelihood of a TB infected individual infecting another person is highest in those with microbiologically confirmed pulmonary TB (PTB) in comparison with other types of TB such as smear-negative pulmonary TB and extra-pulmonary TB [4]. Moreover, the high burden of HIV co-infection results in a rapid increase in the number of tuberculosis patients in the community [4]. Zimbabwe has a high burden of TB/HIV co-infection, which was recorded at 69% in 2013 [1]. In 2013 prevalence estimates of TB morbidity (including HIV/TB co-infections) were 409 (235-630) cases per 100,000 population whilst mortality estimates (including HIV/TB deaths) were 153 (121-189) cases per 100,000 population [1].

Given this, early diagnosis and immediate initiation of TB treatment are therefore essential for an effective TB control programme in order to prevent further disease progression at the individual level and transmission within the community. A systematic review of 52 studies on the length of treatment delays by Sreeramareddy et al [5] showed that average patient delays and health system delays were similar (28.7 versus 25 days) and the trend was similar when stratified into low and high income countries. Other systematic reviews by Storla et al [6] and Finnie et al [7] showed varying factors associated with TB treatment delays but both highlighted the importance of how uptake of measures to shorten these delays may reduce infectious cases and improve TB control. In Finnie et al’s [7] systematic review which was specific to sub-Saharan Africa, consulting traditional leaders first and longer travel time were consistently associated with patient health system delays among other factors. In the Zimbabwe National TB programme, there is insufficient knowledge on the extent of total TB treatment delay and whether such delay is mostly attributable to patient delay or health system delay. The aim of this study was therefore to assess the length of patient and health system delays among TB patients in Zimbabwe and their associated risk factors.

## Methods

### Study design

This was a descriptive cross-sectional study.

### Study participants and sampling

We used a two-stage random sampling design. In the first stage, 4 of the country’s 10 provinces were randomly selected, whilst in the second stage, 3 districts were randomly selected in each province. In the third stage, 4 health facilities were randomly selected from all health facilities that registered ≥30 smear-positive PTB cases per month in each district. In total, 47 health facilities were included in the study of which 3 were provincial hospitals, 15 were district/mission hospitals, 10 were urban clinics and 19 were rural primary health care facilities.

All patients aged ≥ 18 years with microbiologically confirmed PTB and who were started on TB treatment and entered in the TB registers from these facilities between 01 January and 31 March 2013 were sequentially recruited in the study after verbal consent. The diagnosis and confirmation of TB was either by sputum-smear microscopy for acid-fast bacilli or GeneXpert MTB/RIF assay. Using Dobson’s formula, a minimum sample size of 260 study participants was required to estimate the proportion with patient delays >30 days given a 95% confidence interval (CI) with a 5% margin of error, based on 83% of patients experiencing patient delay >30 days as derived from a study by Odusanya et al [8] and an 80% response rate.

### TB diagnosis under the National TB program

In Zimbabwe, TB treatment services are integrated with general health services at all health facilities in the country. When patients present with TB symptoms at a health facility, they are recorded in the TB suspect register and have their sputum examined by direct smear microscopy looking for acid-fast bacilli with a Ziehl-Neelsen stain. Sputum specimens are sent to TB diagnosing centres for sputum-smear microscopy which are often located at selected primary level health facilities (microscopy centres) and all secondary level health facilities (district or mission hospitals) in each district and all tertiary level (provincial) hospitals at the provincial level. In urban areas, TB diagnosing centres are located at selected municipal polyclinics. Smear-positive PTB cases are those with sputum smears positive for acid-fast bacilli [9]. Recently, Gene Xpert is being recommended as the initial TB diagnosis test among presumptive TB patients who are HIV-infected or who have risk factors for drug-resistant TB although it is not available at all TB diagnosing centres. In patients who are known to be HIV-negative and/or who have no risk factors for DR-TB, sputum smear microscopy remains the diagnostic method of choice. Patients with smear-positive sputum or a positive result from Gene Xpert are classified as having microbiologically confirmed PTB and have their results communicated to their respective health facilities by telephone. Upon receiving this communication, these patients are traced in the community by a village health worker (VHW) or an environmental health technician (EHT).

Once traced, these patients are referred back to their nearest health facility where they are registered in the health facility daily observed treatment (DOT) register and are started on anti-TB treatment. All presumptive and confirmed TB patients are offered HIV counselling and testing (opt-out provider-initiated) and those confirmed HIV-positive are provided with [10] HIV treatment and care in the form of cotrimoxazole preventive therapy (CPT) and antiretroviral therapy (ART), provided there is no contra-indication [11].

### Data variables, source of data and data collection

A one-day data collection training exercise was conducted prior to piloting of the questionnaire. Data were collected using a structured questionnaire adapted from WHO [12], and this was administered by selected health workers in the selected health facilities. Variables that were abstracted from TB registers included: date of collection of the sputum specimens, type of TB, name of referring health facility, TB treatment start date, sex and age of the patient. TB registration numbers and TB suspect numbers were collected as patient identifiers in place of patient names. The structured questionnaires were administered to all microbiologically confirmed PTB patients upon commencing TB treatment or during review visits within 1 week of commencing TB treatment. Questionnaires were also translated into the 2 local languages *(Shona and Ndebele)* for those who were not conversant with English. Informed verbal consent was sought prior to the interview and participants were allowed to withdraw from the interviews whenever they pleased.

Variables collected in the questionnaire about timing and treatment delays included:- date of onset of any TB symptoms, date of first encounter with a health worker and number of visits to a health facility prior to starting treatment. Information on the first encounter with a health worker and the number of visits to the health facility was verified wherever possible by looking through the patient’s case notes and their TB treatment card.

### Study definitions

This study focused on *total delay* which was stratified into patient delay and health care system delay. These terms were defined as follows:

#### Patient delay

The time period in days between the onset of TB symptoms and the patient’s first contact with a health worker of >30 days [7,13,14]. Contact with a health worker refers to a nurse or medical doctor at a public health facility (which are the only institutions providing TB treatment) and this excludes pharmacists, private practitioners or traditional healers. Onset of TB symptoms was defined as the first experience of any symptoms suggestive of TB (cough, fever, weight loss, haemoptysis, fatigue, night sweats).

#### Health care system delay

The time period in days between the date of first contact with a health worker and the date of start of TB treatment of >4 days. This is under the assumption that laboratory turn-around times for sputum samples are expected to be less than 72 hours and are coupled with immediate tracing of patients for start of TB treatment for purposes of infection control.

### Statistical analysis

Patient data collected in the structured report form were coded and double entered by three independent data entry clerks into EpiData version 3.1 and later cleaned for errors and analysed using STATA, version 12.1 (Stata Corporation, College Station, Texas). Data were weighted prior to analysis to account for unequal probabilities of selection and to also account for the clustering effect of the sample design. Patient and health care system delays were first summarised and presented as the median (interquartile range) number of days. Patient delay was then categorised as patient delay >30 days whilst health system delay was categorised as health system delay >4 days and these were used for the univariate analysis. Associations between patient characteristics and both types of delay were determined by the chi-square test or alternatively the Fischer’s Exact test if numbers were small. Logistic regression was used to calculate adjusted odds ratios (aOR) and their respective 95% confidence intervals for factors associated with patient delay and health system delay. All patient characteristics with p-values < 0.25 or those thought to be biologically plausible were included in both the univariate and multivariate logistic regression models. P-values of less than 0.05 were considered statistically significant.

### Ethics and approval

Ethics approval was obtained locally from the Medical Research Council of Zimbabwe (MRCZ) and also the Ethics Advisory Group (EAG) for the International Union Against Tuberculosis and Lung Disease (IUATLD).

## Results

### Patient characteristics

During the 3-month study period, all 383 interviewed patients gave voluntary consent to participate in the survey of whom the majority, 211 (55%) were male (Table 1). Overall median age of study participants was 34 years (IQR, 28-43). Most patients (62%) were recruited upon starting TB treatment from either district or mission hospitals. The majority of these patients, 280 (75%), started TB treatment at TB diagnosing centres were they had also been diagnosed. Tuberculosis diagnosis was mainly by sputum-smear microscopy (95%). Nearly all patients (98%) had been HIV-tested: there were 230 (62%) who were HIV-infected. Most patients (70%) resided in rural areas, unemployment was high (62%) and more than half (55%) had attained secondary school education or higher.

**Table 1:** **Characteristics of tuberculosis patients in Zimbabwe**

**Characteristics (N = 383)**	**n**	**N**	**Weighted percent (95% CI)**
*Age in years*			
18 – 25	53	383	13.9% (10.5-18.2)
26 – 44	244	383	63.8% (58.3-69.0)
45 – 54	54	383	14.2% (10.6-18.6)
≥55	31	383	8.1 (5.7-11.6)
*Sex*			
Female	172	383	45.0% (39.5-50.6)
Male	211	383	55.0% (49.4-60.5)
*Type of diagnostic test*			
Sputum microscopy	363	383	94.8% (93.0-96.2)
Gene Xpert	20	383	5.2% (3.8-7.0)
*Type of health facility*			
Provincial hospital	18	383	4.7% (3.1-6.9)
District/mission hospital	237	383	61.8% (58.5-64.9)
Urban municipal	73	383	19.1% (16.7-21.8)
Rural primary healthcare facility	55	383	14.5% (11.4-18.2)
*Diagnosing centre*			
Same as DOT facility	280	373	75.1% (71.8-78.2)
Different from DOT facility	93	373	24.9% (21.8-28.3)
Missing	10	383	2.6%
*HIV tested*			
No	6	376	1.6% (0.8-3.4)
Yes	370	376	98.4% (96.6-99.2)
Missing	7	383	1.9%
*HIV status*			
HIV-negative	140	370	37.9% (32.6-43.6)
HIV-positive	230	370	62.1% (56.4-67.4)
*HIV + ve patient on CPT/ART prior to TB treatment*			
No	72	230	31.2% (25.4-37.6)
Yes	158	230	68.8 (62.4-74.7)
*Staying with anyone currently on TB treatment*			
No	348	383	90.9% (86.3-95.3)
Yes	35	383	9.1% (6.3-13.1)
*Education*			
More than secondary	20	382	5.3% (3.3-8.4)
Secondary	192	382	50.3% (45.0-55.6)
Primary	145	382	38.0% (33.1-43.1)
No education	25	382	6.5% (4.2-9.8)
Missing	1	383	0.4%
*Employment status*			
Formal employment	40	379	10.7% (8.0-14.2)
Informal employment	105	379	27.6% (23.1-32.6)
Unemployed	234	379	61.8% (56.7-66.6)
Missing	4	383	1.0%
*Residence*			
Urban	114	378	30.1% (26.1-34.5)
Rural	264	378	69.9% (65.6-73.9)
Missing	5	383	1.3%
*History of smoking*			
Never smoked	269	380	70.7% (65.3-75.5)
Current smoker	26	380	6.8% (4.3-10.6)
Quit smoking	86	380	22.5% (18.3-27.4)
Missing	3	383	0.8%
*History of alcohol use*			
Never	213	373	57.0% (51.4-62.4)
Current drinker	29	373	7.7% (5.5-10.6)
Quit alchohol use	132	373	35.3% (30.2-40.9)
Missing	10	383	2.6%
*Previous contact with TB patient*			
No	210	383	54.8% (48.4-62.1)
Yes	173	383	45.2% (39.8-50.6)
*Marital status*			
Married	225	380	59.3% (53.6-64.7)
Single	67	380	17.6% (13.7-22.4)
Divorced/separated	35	380	9.3% (6.6-12.9)
Widowed	52	380	13.8% (10.2-18.4)
Missing	3	383	0.8%
*Took self medication from pharmacy*			
No	335	383	87.5% (84.0-90.3)
Yes	48	383	12.5% (9.7-16.0)
*Took traditional medicine*			
No	348	383	90.8% (87.3-93.4)
Yes	35	383	9.2% (6.6-12.7)
*Visited a private practitioner*			
No	354	383	92.4% (89.4-94.7)
Yes	29	383	7.6% (5.4-10.6)
*Health care visits prior to diagnosis*			
1	173	378	45.6% (40.2-51.1)
2	121	378	31.9% (26.9-37.3)
3	40	378	10.5% (7.6-14.4)
≥4	45	378	12.0% (9.1-15.7)
Missing	5	383	1.2%
*Time taken to reach health facility*			
<30 minutes	150	377	39.9% (35.1-44.8)
30mins - 1 hour	101	377	26.7% (21.9-32.0)
>1 hour	126	377	33.5% (28.6-38.8)
Missing	6	383	1.5%
*Distance to health facility(in km)*			
≤5	122	331	36.8% (32.3-41.6)
6 – 15	64	331	19.5% (15.4-24.3)
16 – 30	55	331	16.5% (12.3-21.8)
≥30	90	331	27.2% (22.4-32.5)
Missing	52	383	13.7%
*Difficulty in accessing transport to health centre*			
No	322	383	84.2% (78.9-89.5)
Yes	61	383	15.8% (12.1-20.5)
*Ever heard of TB before diagnosis*			
Yes	43	365	88.1% (83.6-91.5)
No	322	365	11.9% (8.5-16.4)
Missing	18	383	4.8%

NB: Percentages may not always add up to 100 because of rounding off error.

DOT = directly observed treatment; HIV = human immunodeficiency virus; CPT = cotrimoxazole preventive therapy; ART = antiretroviral therapy, TB = tuberculosis.

### Patient delays and associated factors

There was a median of 28 days for patient delays (IQR, 21-63). Of all recruited study participants, 184 (48%) experienced patient delays >30 days in seeking TB treatment services.

Table 2 shows factors associated with patient delay. In comparison to patients starting TB treatment at district or mission hospitals, those starting treatment at rural primary health facilities were more likely [adjusted odds ratio (aOR) 2.70, 95% confidence interval (CI) 1.27-5.75, p = 0.01] to experience patient delays. On further analysis, those patients who started TB treatment at rural facilities accessed traditional healers first more than those accessing other types of health facilities although the association was not statistically significant (14.2% vs. 8.4%, p = 0.192). The seeking of treatment first at private practitioners was also similar for both those who were treated at rural facilities and other types of health facilities (7.8% vs. 7.5%, p = 0.937).

**Table 2:** **Factors associated with patient delay in accessing TB treatment services**

**Characteristic (N = 383)**		Patient delay > 30 days*	
	**n (%)**	**OR (95% CI)**	**Adjusted OR (95% CI)**
*Age in years*			
18 – 25	30 (55.8)	Reference	Reference
26 – 44	122 (50.0)	0.79 (0.41-1.53)	0.55 (0.25-1.19)
45 – 54	24 (43.9)	0.62 (0.26-1.46)	0.55 (0.21-1.43)
≥55	9 (29.4)	0.33 (0.12-0.88)	0.35 (0.10-1.24)
*Sex*			
Female	80 (46.5)	Reference	Reference
Male	105 (49.7)	1.13 (0.72-1.78)	1.07 (0.59-1.96)
*Type of health facility*			
District/mission hosp	95 (51.2)	Reference	Reference
Provincial hospital	10 (5.2)	1.77 (0.64-4.86)	1.82 (0.53-6.30)
Urban municipal clinic	45 (24.6)	2.45 (1.42-4.22)	1.78 (0.88-3.60)
Rural primary healthcare facility	35 (19.0)	2.61 (1.36-5.01)	2.70 (1.27-5.75)
*HIV status*			
Not tested	2 (40.0)	Reference	Reference
HIV negative	63 (44.6)	1.21 (0.25-5.81)	1.01 (0.16-6.51)
HIV positive, not on ART	36 (51.0)	1.56 (0.32-7.66)	1.38 (0.22-8.77)
HIV positive, on ART/CPT	80 (50.5)	1.53 (0.32-7.30)	1.68 (0.26-10.77)
*Staying with anyone currently on TB treatment*			
No	170 (48.9)	Reference	Reference
Yes	15 (41.7)	0.75 (0.35-1.61)	0.99 (0.79-1.23)
*Education*			
More than secondary	10 (48.2)	Reference	Reference
Secondary	101 (52.7)	1.20 (0.43-3.31)	1.40 (0.45-4.37)
Primary	66 (44.8)	0.87 (0.31-2.45)	1.35 (0.39-4.74)
No education	9 (36.7)	0.62 (0.17-2.29)	1.10 (0.23-5.33)
*Employment status*			
Formal employment	25 (61.2)	Reference	Reference
Informal employment	54 (51.5)	0.67 (0.33-1.39)	0.92 (0.37-2.27)
Unemployed	104 (44.3)	0.50 (0.26-1.00)	0.74 (0.30-1.85)
*Residence*			
Urban	60 (52.8)	Reference	Reference
Rural	119 (46.2)	0.82 (0.53-1.26)	1.04 (0.80-1.33)
*History of smoking*			
Never	131 (48.8)	Reference	Reference
Current smoker	10 (36.8)	0.61 (0.24-1.57)	0.59 (0.20-1.78)
Quit smoking	43 (50.3)	1.06 (0.63-1.80)	0.75 (0.35-1.62)
*History of alcohol use*			
Never	98 (46.2)	Reference	Reference
Current drinker	12 (43.0)	0.88 (0.40-1.91)	1.19 (0.39-3.57)
Quit alcohol use	69 (52.3)	1.28 (0.78-2.08)	1.26 (0.61-2.64)
*Previous contact with TB patient*			
No	100 (47.8)	Reference	Reference
Yes	84 (48.8)	1.03 (0.66-1.59)	0.96 (0.79-1.16)
*Took traditional medicine*			
No	163 (46.8)	Reference	Reference
Yes	22 (62.4)	1.88 (0.84-4.20)	1.80 (0.76-4.28)
*Took self-medication from pharmacy*			
No	140 (44.5)	Reference	Reference
Yes	45 (65.4)	2.35 (1.35-4.11)	2.33 (1.23-4.43)
*First consulted private practitioner*			
No	166 (46.9)	Reference	Reference
Yes	19 (65.3)	2.14 (0.95-4.81)	1.47 (0.56-3.87)
*Time taken to reach health facility*			
<30 minutes	89 (59.3)	Reference	Reference
30mins - 1 hour	40 (39.9)	0.45 (0.26-0.81)	0.43 (0.22-0.83)
>1 hour	54 (42.5)	0.51 (0.31-0.82)	0.47 (0.24-0.94)
*Distance to health facility (in km)*			
≤5	66 (54.1)	Reference	Reference
6 – 15	28 (43.8)	0.66 (0.36-1.21)	1.14 (0.52-2.47)
16 – 30	27 (50.1)	0.85 (0.41-1.76)	1.85 (0.79-4.37)
≥30	39 (43.0)	0.64 (0.35-1.18)	1.39 (0.60-3.23)
*Difficulty in accessing transport to health centre*			
No	150 (46.6)	Reference	Reference
Yes	35 (57.3)	1.51 (0.80-2.85)	0.66 (0.40-1.08)
*Ever heard of TB before diagnosis?*			
No	18 (40.4)	Reference	Reference
Yes	156 (48.7)	1.40 (0.63-3.13)	1.16 (0.97-1.40)
**Total**	184 (48.3)	-	-

*Patient delay was defined as a period >30 days from onset of any TB symptom to date of first encounter with health worker.

HIV = human immunodeficiency virus; CPT = cotrimoxazole preventive therapy; ART = antiretroviral therapy; TB = tuberculosis; VCT = voluntary counseling and testing; OR = odds ratio; CI = confidence interval.

The likelihood of patient delays was higher for those who took self medication when experiencing cough symptoms [aOR 2.33, 95% CI 1.23-4.43, p = 0.01]. Those taking between 30 minutes to an hour to reach their DOT facility [aOR 0.43, 95% CI 0.22-0.83, p = 0.013] and >1 hour [aOR 0.47, 95% CI 0.24-0.94, p = 0.032] were both less likely to experience patient delays when compared to those reaching the DOT facility in less than 30 minutes.

### Health system delays and associated factors

There was a median of 2 days for health system delays (IQR, 1-5). There were only 118 (31%) of all study participants who encountered health system delays >4 days. Table 3 shows factors associated with health system delay >4 days. Health system delays were higher among those 26-44 years old [aOR 3.30, 95% CI 1.16-9.37, p = 0.025] and aged 45-54 years [aOR 3.57, 95% CI 1.09-11.72, p = 0.036]. Being male and having TB diagnosis done using GeneXpert were also associated with less odds of health system delays i.e. [aOR 0.47, 95% CI 0.23-0.96, p = 0.038] and [aOR 0.21, 95% CI 0.07-0.66, p = 0.008] respectively. All those who had a GeneXpert test done were HIV-infected individuals.

**Table 3:** **Factors associated with Health system delays in accessing TB treatment services**

**Characteristic (N = 383)**		Health system delay > 4 days*	
	**n (%)**	**OR (95% CI)**	**Adjusted OR (95% CI)**
*Age in years*			
18 – 25	9 (17.3)	Reference	Reference
26 – 44	84 (34.4)	2.51 (1.08-5.84)	3.30 (1.16-9.37)
45 – 54	18 (32.4)	2.30 (0.85-6.22)	3.57 (1.09-11.72)
≥55	7 (20.9)	1.26 (0.38-4.18)	2.66 (0.61-11.57)
*Type of diagnostic test*			
Direct smear microscopy	110 (30.4)	Reference	Reference
Gene Xpert	7 (35.6)	1.27 (0.56-2.85)	0.21 (0.07-0.66)
*Sex*			
Female	61 (35.6)	Reference	Reference
Male	56 (26.6)	0.66 (0.41-1.06)	0.47 (0.23-0.96)
*Type of health facility*			
District/mission hospital	48 (20.5)	Reference	Reference
Provincial hospital	12 (64.6)	7.08 (2.81-17.88)	14.15 (4.58-43.69)
Urban municipal	37 (50.1)	3.89 (2.21-6.86)	6.9 (2.41-19.82)
Rural primary healthcare facility	21 (37.6)	2.33 (1.19-4.58)	2.67 (1.18-6.07)
*TB Diagnosing centre on site*			
Yes	71 (25.5)	Reference	Reference
No	43 (45.8)	2.48 (1.53-4.02)	1.41 (0.59-3.36)
*HIV status*			
Not tested	2 (33.5)	Reference	Reference
HIV-negative	38 (27.0)	0.73 (0.13-4.11)	0.72 (0.06-8.97)
HIV-positive, not yet on ART	20 (28.0)	0.77 (0.13-4.52)	0.70 (0.06-8.99)
HIV-positive, on ART/CPT	55 (34.5)	1.04 (0.19-5.78)	1.37 (0.11-17.04)
*Residence*			
Urban	43 (37.6)	Reference	Reference
Rural	74 (27.9)	0.81 (0.54-1.22)	0.95 (0.71-1.29)
*History of smoking*			
Never	80 (29.9)	Reference	Reference
Current smoker	7 (25.1)	0.79 (0.29-2.14)	1.58 (0.45-5.63)
Quit smoking	29 (34.2)	1.22 (0.70-2.12)	0.86 (0.39-1.89)
*History of alcohol use*			
Never	64 (30.1)	Reference	Reference
Current drinker	5 (18.6)	0.53 (0.20-1.37)	0.40 (0.11-1.42)
Quit alcohol use	46 (34.7)	1.23 (0.74-2.05)	1.50 (0.69-3.26)
*Took traditional medicine*			
No	109 (31.3)	Reference	Reference
Yes	9 (24.6)	0.72 (0.31-1.65)	0.66 (0.28-1.57)
*Took self-medication from pharmacy*			
No	98 (29.2)	Reference	Reference
Yes	20 (41.3)	1.71 (0.90-3.23)	1.68 (0.72-3.93)
*Health care visits prior to diagnosis*			
1	46 (26.8)	Reference	Reference
2	40 (33.5)	1.38 (0.79-2.41)	1.82 (0.93-3.53)
3	10 (25.2)	0.92 (0.39-2.15)	0.99 (0.33-2.96)
≥4	20 (43.9)	2.14 (1.06-4.34)	2.24 (1.02-4.94)
*Time taken to reach health facility*			
<30 minutes	52 (34.3)	Reference	Reference
30mins - 1 hour	33 (33.1)	0.95 (0.52-1.71)	1.38 (0.65-2.93)
>1 hour	32 (25.4)	0.65 (0.37-1.15)	1.03 (0.46-2.27)
*Distance to health facility (in km)*			
≤5	44 (36.4)	Reference	Reference
6 – 15	17 (26.7)	0.64 (0.32-1.27)	0.78 (0.32-1.88)
16 – 30	18 (32.9)	0.86 (0.41-1.78)	1.84 (0.68-4.98)
≥30	15 (16.5)	0.35 (0.16-0.75)	0.51 (0.20-1.29)
*Difficulty in accessing transport to health centre*			
No	21 (32.6)	Reference	Reference
Yes	96 (30.3)	1.14 (0.60-2.19)	1.00 (0.64-1.57)
**Total**	118 (30.7)	-	-

*Health system delay was defined as a period >4 days from first encounter with health worker to date of starting TB treatment.

DOT = directly observed treatment; HIV = human immunodeficiency virus; CPT = cotrimoxazole preventive therapy; ART = antiretroviral therapy; TB = tuberculosis, VCT = voluntary counseling and testing.

In addition, the odds of health system delays were higher when starting treatment at urban municipal clinics [aOR 6.9, 95% CI 2.41-19.82, p = 0.03] and rural clinics [aOR 2.67, 95% CI 1.18-6.07, p = 0.019] although these delays were not significantly higher for those accessing treatment from non-TB diagnosing facilities after multivariate analysis. Lastly, there were higher odds of health system delays associated with encountering ≥4 health care visits in comparison to one healthcare visit prior to starting TB treatment [aOR 2.24, 95% CI 1.02-4.94, p = 0.045]. Further stratification of those with ≥4 healthcare visits by type of health-worker seen among those experiencing health system delays showed that 8 (42%) had seen different health workers in the same facility and 11 (58%) had seen different health workers in different health facilities.

### Total delays

**Figure 1:**
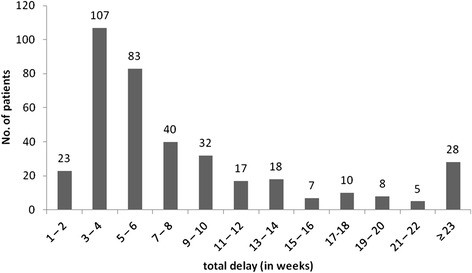
**Total delay in weeks between onset of TB-related symptoms and treatment of TB.**

Total delay among all recruited study participants was a median of 36 days (IQR, 23-68). Figure 1 shows the distribution of total delays in weeks.

## Discussion

In this study, we assessed the extent of both patient and health system delays in accessing TB treatment within public health facilities under the Zimbabwe National Tuberculosis Programme. The median patient delay was 28 days and this is similar to findings from other African countries such as Ghana [15] and South Africa [16] and slightly lower than reported findings from other low income countries as reported in a systematic review by Sreeramareddy et al [5]. The taking of self-medication for TB-related symptoms, as has been found in other studies [17,18], was associated with patient delay, although in general this practice was uncommon amongst this patient population. Also uptake of traditional medicine was not significantly associated with patient or health system delays and assuming there was no under-reporting, this practice was uncommon despite the fact that more than two thirds of study participants resided in a rural setting. This finding differs from findings from a meta-analysis by Finnie et al [7] of other sub-Saharan African countries where taking of traditional medicine was consistently found to be associated with patient delays.

Patients who started TB treatment at rural primary healthcare facilities were more likely to experience patient delays when compared to those who started at district or mission hospitals. Literature from other countries also indicates that an initial visit to a government low-level health care facility [19-21] and rural residence [14,16,22] are both associated with increased patient delays in seeking TB treatment services. Whilst the majority of our study participants were rural patients, distance to a DOT facility was not associated with patient delay and in fact those taking a longer time to reach a DOT facility were less likely to experience patient delays. The reasons for this finding are not clear as other studies have found that long distances to health facilities [7] and absence of transport have contributed to poor and delayed uptake of HIV care and treatment in co-infected patients [23].

It is likely that the patient delays among those with shorter travel time to a health facility and accessing treatment at lower-level health facilities are attributed to these patients being less health conscious and seeking treatment late in comparison to those who are more health conscious who seek services sooner at higher-level hospitals even though they may be further away. Zimbabwe has a four-tiered referral health system and district/mission hospitals which are at the second level may be preferable to patients since they manage referral cases not managed at primary care facilities which are at the first level, as they have a staff complement of medical doctors in addition to nursing staff. They also provide specialized services to periphery first-level health facilities within their district such as laboratory services and ART initiation prior to decentralization of ART initiations to lower-level health facilities. These district/mission hospitals are also centrally located within the district and therefore usually better resourced and managed and generally further away from patients in comparison to primary facilities, particularly in rural settings.

Contrary to other published studies, gender [24], education level [13] and old age [25,26] were not associated with patient delays. In our study, HIV status showed no association with patient or health system delays, as has been found elsewhere [16,27].

In this study, the greater proportion of delay in seeking TB treatment was a result of patient-related factors rather than health system factors, a finding that is similar to that of other countries [8,17,28,29]. The median health system delay was just 2 days, which is low in comparison to findings from other studies done in both intermediate [30,31] and high-burden TB [14,16,19] settings. This encouraging finding highlights the efficiency within the national TB programme of processes that exist from sputum collection to sputum examination to diagnosis and the return of results to the health worker and eventual transmission of information to the patient which is followed by the initiation of the patient on anti-TB treatment among microbiologically confirmed PTB cases.

Of note were the higher health system delays among those receiving treatment at lower-level health facilities and those with four or more health facility visits prior to starting TB treatment. Primary health-care facilities usually do not have TB diagnosing facilities on site and therefore refer sputum samples to TB diagnosing centres at second-level health facilities thereby increasing sputum turnaround times, especially in rural settings. Whilst sputum transportation using motorbikes has been established in some districts ranging from 2-day to 1-week intervals, it remains ad-hoc in some places and this needs improvement. In Zimbabwe, Gene Xpert which is a more accurate and rapid diagnostic test [32], has recently been recommended [33] and piloted as the initial diagnostic test for HIV-infected persons in addition to those with drug resistant TB risk factors as recommended by WHO [34]. This explains why fewer health system delays were noted for those HIV-infected patients with TB diagnosed using the Gene Xpert MTB/RIF assay compared to the conventional smear microscopy.

Those who had four or more visits had either seen different health workers in the same facility or different health workers in different health facilities and this finding may highlight an important barrier to early diagnosis and treatment of TB, especially among mobile populations where each consecutive visit to a different health care worker may be like a first time visit if the hospital or patient records are not properly scrutinised. This calls for an important need to strengthen TB case diagnosis and management trainings among health workers to ensure that information about investigations and management is effectively passed on from one health care worker to another. In general, however, the majority of patients (78%) had visited the health facility ≤ 2 times before the diagnosis of TB was made, and this does suggest that the awareness and knowledge of TB is high among health workers in a high HIV-TB burden country like Zimbabwe. This high awareness may relate to the high burden of disease in Zimbabwe: in 2008 alone, TB cases contributed 30% of all chronic out-patient disease conditions [35].

Unlike other studies done in urban settings [13,36,37] our study includes a heterogenous population of TB patients in Zimbabwe coming from different levels of the healthcare system, from different regions and with a rural and urban mix, and therefore is representative of the overall TB programme in Zimbabwe. Limitations of our study include a possibility of recall bias of past events among respondents relating to when they first encountered TB symptoms. Health system delays may have shortened during the time of the survey since the structured questionnaires were administered by health workers from the selected health facilities. Our study could have failed to capture information on confirmed TB cases that were started on TB treatment but were not registered as shown in a study conducted in one province of Zimbabwe in which 43% of treated TB cases were not registered [38].

There is also a possibility that our patient sample excluded vital information on those patients who may have been suspected of TB or diagnosed of TB in the private sector but did not go to public health facilities despite being referred. This is given the background that in Zimbabwe, TB treatment services are only offered in public health facilities hence those that are suspected of TB or diagnosed with TB in private health care facilities are referred to public health facilities which could either be missionary, local authority or government health facilities.

## Conclusion

This study has assessed the extent of TB treatment delays in a country with a high HIV-TB burden, an important issue that relates to individual patient outcomes and TB transmission in the community. Patient delays were found to be moderately high, suggesting the need for increased advocacy, communication and social mobilization efforts targeted at informing the general population to seek health services early when they experience symptoms suggestive of TB. There is also need for engagement and training of pharmacy staff so that they can accurately identify people with TB-like symptoms and effectively explain to them that TB diagnosis and treatment are offered for free at public health institutions. A programme of this nature has been piloted in Cambodia and has shown positive results [39].

On the other hand, health system delays were not common, suggesting an efficient TB programme. Better understanding about why some patients have repeated visits to health facilities is needed. We also recommend more qualitative studies to elicit patient responses on why patient delays were associated with access to rural primary health facilities and also why health system delays where linked to older age and being female. Whilst our study findings are generalizable to those patients with microbiologically confirmed PTB, they are not representative of the HIV patient population which largely has smear negative TB and among whom delays are likely to be much more complex, longer, and more difficult to address. Hence a study specific to this population is also necessary.
